# Why do prospective and retrospective measures of childhood maltreatment differ? Qualitative analyses in a cohort study

**DOI:** 10.1016/j.chiabu.2024.107070

**Published:** 2024-10-03

**Authors:** Oonagh Coleman, Jessie R. Baldwin, Terrie E. Moffitt, Louise Arseneault, Helen L. Fisher, Kelly Rose-Clarke, Andrea Danese

**Affiliations:** a Social, Genetic and Developmental Psychiatry Centre, Institute of Psychiatry, Psychology & Neuroscience, King’s College London, London, UK; b Department of Clinical, Educational and Health Psychology, Division of Psychology and Language Sciences, University College London, London, UK; c Department of Psychology and Neuroscience, Duke University, Durham, NC, USA; d ESRC Centre for Society and Mental Health, King’s College London, London, UK; e Department of Global Health and Social Medicine, King’s College London, London, UK; f Department of Child and Adolescent Psychiatry, Institute of Psychiatry, Psychology & Neuroscience, King’s College London, London, UK; g National and Specialist CAMHS Clinic for Trauma, Anxiety, and Depression, South London and Maudsley NHS Foundation Trust, London, UK

**Keywords:** Childhood trauma, Maltreatment measurement, Maltreatment disclosure

## Abstract

**Background:**

Research indicates that prospective and retrospective measures of maltreatment often identify different groups of individuals, yet the reasons for these discrepancies remain understudied.

**Objective:**

This study explores potential sources of disagreement between prospective and retrospective measures of maltreatment, utilising qualitative data from interviewers’ notes.

**Participants and setting:**

The Environmental Risk Longitudinal Twin Study includes 2232 children followed from ages 5–18. Prospective measures relied on caregiver interviews and researcher observations from ages 5–12, while retrospective measures involved self-reports via the Childhood Trauma Questionnaire at age 18.

**Methods:**

We purposively sampled written interviewer notes from 36 participants who reported more types of maltreatment retrospectively than prospectively (‘new reports’ group) and 31 participants who reported fewer types retrospectively than prospectively (‘omitted reports’ group). We conducted a framework analysis of the notes, comparing between the two groups to explore explanations for measurement disagreement.

**Results:**

Three categories of themes emerged related to measurement discrepancies: challenges with prospective measures, highlighting reasons given by the ‘new reports’ group for why maltreatment went undetected or was not adequately responded to prospectively; challenges with retrospective measures that highlight difficulties with openness and accuracy of self-reports; and differences in appraisals of violence or distressing childhood experiences between the two groups that might lead to new or omitted retrospective reports.

**Conclusions:**

Our findings underscore potential mechanisms underlying the disagreement between prospective and retrospective measures, contributing to better understanding of these different constructs and more balanced interpretation of related findings.

## 1. Introduction

Child maltreatment has been identified as an important risk factor for poor mental and physical health, including depression (Brown et al., 1999), behavioural problems (Widom, 1989), psychotic symptoms (Arseneault et al., 2011), and obesity (Danese and Tan, 2014). Identifying individuals exposed to maltreatment is therefore key to mapping underlying pathophysiological mechanisms and mitigating health consequences. However, the way in which maltreatment is defined, identified, and measured, has drawn considerable research attention (Danese, 2020; Kendall-Tackett and Becker-Blease, 2004; Widom et al., 2004).

Child maltreatment can be measured prospectively and retrospectively. These measures differ in two key ways. Firstly, the measurement timing: prospective measures are collected during childhood, while retrospective measures are collected in adulthood. Secondly, the measurement source: prospective measures typically rely on informant reports (e.g., parent) or official records (e.g., child protection records), while retrospective measures and are typically self-reported, capturing first-person, subjective appraisals and memories. Meta-analytic findings suggest that prospective and retrospective measures identify different groups of individuals and therefore should not be used interchangeably (Baldwin et al., 2019).

Several mechanisms could contribute to the poor agreement between prospective and retrospective measures of maltreatment (Baldwin et al., 2024) including systematic differences between measures such as the reporter (Fisher et al., 2011; Hardt and Rutter, 2004), measurement type and sensitivity (e.g., interview, questionnaire, or official record) (Danese and Widom, 2020), or period of observation (Reuben et al., 2016). However, even when studies use repeated self-report measures at both time-points and use the same observational period, measurement of maltreatment has poor intra-rater reliability (Colman et al., 2016; Langeland et al., 2015). This suggests measurement issues cannot fully account for disagreement between prospective and retrospective measures.

Beyond measurement issues, two explanations for disagreement between measures have been proposed: memory mechanisms and motivational factors (Baldwin et al., 2019; Coleman et al., 2024; Danese, 2020). Memory mechanisms refer to processes involved in the formation and recall of memories that can lead to retrospective under- and over-reporting. These reports depend on experiences being encoded and consolidated into long-term memory and being accessible during retrospective measurement. Factors such as appraisals at the time of encoding and recall, biases or distortions in existing memories, and cognitive strategies to avoid recalling distressing experiences can all influence these reports (Goodman et al., 2010; Henry et al., 1994; Susser and Widom, 2012). Motivational factors are potential incentives informants might have to withhold or fabricate information about an abusive experience (Baldwin et al., 2019; Femina et al., 1990; Paine and Hansen, 2002). These include internal factors, such as shame or guilt, and interpersonal dynamics that affect reporting. These motivations may be intentional, where individuals are aware of how they are influencing their decision to disclose, or unintentional, influencing behaviour outside of their awareness.

This study seeks to explore potential sources of disagreement between prospective and retrospective measures of maltreatment, using qualitative data from a longitudinal cohort study in the UK. Previous research in this cohort revealed low agreement between individuals identified as maltreated by these different measures (Cohen’s Kappa coefficient of agreement of 0.19) (Newbury et al., 2018). To explore reasons for the discrepancy, we analysed detailed written notes from interviewers reflecting on the retrospective interviews they conducted with participants at age 18. The notes shed light on the interview setting, interpersonal relations between interviewer and participant, and how participants navigate and communicate their childhood experiences.

## 2. Methods

### 2.1. Study design

We analysed qualitative data from the Environmental Risk (E-Risk) Longitudinal Twin Study, which tracks the development of 2232 British twin children born in England and Wales in 1994–95.

### 2.2. E-Risk procedures

The original E-Risk sample was constructed in 1999–2000, when 1116 families (93 % of those eligible) with same-sex 5-year-old twins participated in home visit assessments. This sample comprised 56 % monozygotic and 44 % dizygotic twin pairs; sex was evenly distributed within zygosity (49 % male); 90 % identified as white. Full details of the sample are reported elsewhere (Moffitt and the E-Risk Study Team, 2002). The Joint South London and Maudsley and the Institute of Psychiatry Research Ethics Committee approved each phase of the study.

Follow-up home visits were conducted when the children were aged 7 (98 % participation), 10 (96 %), 12 (96 %), and 18 (93 %). At age 18, 2066 participants took part in the assessments. The average age of the twins at this time was 18.4 years (*SD* = 0.36); all interviews were conducted after their 18th birthday. There were no differences between those who did and did not take part at age 18 in terms of socioeconomic status (SES) assessed when the cohort was initially defined (χ^2^ = 0.86, *p* = 0.65), age-5 IQ scores (*t* = 0.98, *p* = 0.33), age-5 behavioural (*t* = 0.40, *p* = 0.69) or emotional problems (*t* = 0.41, *p* = 0.68), or childhood poly-victimization (*z* = 0.51, *p* = 0.61). The sample represents the socioeconomic conditions in Great Britain, as reflected in the families’ distribution on a neighbourhood-level socioeconomic index (ACORN [A Classification of Residential Neighbourhoods], developed by CACI Inc. for commercial use) (Odgers et al., 2012). Supplementary Fig. 1 shows *E*-Risk families’ addresses are a near-perfect match to the deciles of the UK’s 2015 Lower-layer Super Output Area (LSOA) Index of Multiple Deprivation (IMD) which averages 1500 residents; approximately 10 % of the cohort fills each of IMD’s 10 % bands, a near-perfect match to the population.

Home visits at 5, 7, 10, and 12 years included assessments with participants and their mother or an alternative primary caregiver. The home visit at age 18 included interviews only with the participants. Study interviewers were psychology graduates or nurses. Data were collected on topics including mental and physical health, school performance, victimization, family and neighbourhood environment, and biomarkers.

The age-18 assessment was undertaken by 14 female interviewers. Prior to data collection, interviewers undertook intensive training for around four weeks in which they were instructed on interview technique, administering measures, making observations, and ethical issues. Interviewers were only involved in data collection after receiving accreditation and approval from the project leads. Interviewers were informed when participants had a history of victimization detected during previous stages of the study.

### 2.3. Assessment of maltreatment in E-Risk

Maltreatment was assessed prospectively while the participants were growing up and retrospectively by asking the participants to reflect on their childhood.

#### 2.3.1. Prospective measures of maltreatment

Exposure to several types of maltreatment was assessed prospectively when participants were aged 5, 7, 10, and 12 (assessment at age 5 concerned maltreatment since birth), from 1999 to 2007. Interviewers visited the home in pairs and were trained to detect signs of abuse or neglect. During each visit, interviewers interviewed the primary caregiver (usually the mother) using a structured interview about child harm, tested the participants, and observed the family environment for evidence of neglect using the Home Observation for Measurement of the Environment (HOME) (Bradley and Caldwell, 1977). Caregivers were asked several questions about whether either of their twins had been intentionally harmed (physically or sexually) by an adult or had contact with welfare agencies, with the focus being on the nature of the maltreatment rather than the perpetrator’s identity. If caregivers endorsed a question, follow-up questions were asked. Interviewers took extensive notes on what had happened and whether the participant had been physically and/or psychologically harmed.

Comprehensive dossiers were compiled for each participant with cumulative information about exposure to physical abuse by an adult; sexual abuse; physical neglect; and emotional abuse/neglect. The dossiers consisted of reports from caregivers on maltreatment, recorded narratives of the interviews with caregivers, recorded debriefings with interviewers who had coded any indication of abuse and neglect at any of the home visits, and information from clinicians when the study team made a child-protection referral. The dossiers were reviewed by two independent researchers and rated for the presence and severity (none/mild/severe) of each type of maltreatment (see Supplementary Material for further information). Inter-rater agreement between the coders exceeded 85 % among the maltreatment cases, and discrepancies between raters were resolved by consensus review.

As in previous E-Risk papers (Latham et al., 2021; Newbury et al., 2018) this study used a dichotomized version of each type of prospectively measured maltreatment which separated maltreatment scores into none/mild (0), versus severe (1).

#### 2.3.2. Retrospective measures of maltreatment

Maltreatment was measured retrospectively using the Childhood Trauma Questionnaire (CTQ; Bernstein and Fink, 1998) when participants were aged 18, from 2012 to 2013. The CTQ is a 25-item questionnaire used for retrospective recall of five forms of maltreatment and has high inter-rater reliability and construct and convergent validity (Fink et al., 1995). Participants reported on their personal experiences of physical, sexual, and emotional abuse, and physical and emotional neglect for the period before they were 12 years old (i.e., before entering secondary school). Almost all (99.5 %; *N* = 2055) participants who took part in the age-18 assessment completed the CTQ.

Maltreatment scores were dichotomized following CTQ guidelines (Bernstein and Fink, 1998) to represent none/low (0) versus moderate/severe (1) maltreatment. Because the prospective measures described above combined scores for emotional abuse and neglect, the CTQ scores for emotional abuse and emotional neglect were also combined for the analyses to allow a direct comparison between prospective and retrospective measures.

### 2.4. Qualitative data collection

Lined sections for text notes were interspersed with the questions in the interviewer impressions section for the age-18 assessment. Interviewers were instructed to write notes after the interview and to allocate ample time for this to record as much as possible. The objective was to document, in a free form way, what the interviewer perceived to be the most salient contextual details of the interview and impressions of the subject. Notes were subsequently transcribed to electronic format. The length of notes varied, and cases with complex life histories tended to have longer notes.

### 2.5. Sampling strategy for qualitative analysis

We categorized participants based on how much their prospective and retrospective measures of maltreatment differed. For each type of maltreatment (physical abuse; sexual abuse; physical neglect; emotional abuse/neglect) we allocated a score of ‘one’ where it was present. A participant with all four types of maltreatment received a score of four; those with only one type scored one. The prospective score and the retrospective scores ranged from zero to four. We subtracted each participant’s prospective score from their retrospective score to create a measure of directional divergence ranging from minus four to plus four. Table 1 shows participants categorized by maltreatment measure discrepancy score.

Notes from interviews with participants with difference scores of two or more (i.e., scores equal or greater than two or equal or lower than minus two) were selected for the qualitative analysis (see Table 1). This a-priori cut-off was established with the expectation that focusing on notes from interviews with participants with the most discrepant maltreatment reports would provide a rich dataset for exploring measurement discrepancies.

For the purpose of the qualitative analysis, we termed participants with greater retrospective than prospective scores (i.e., participants who self-reported forms of maltreatment that were not prospectively detected) the *‘new reports’* group. Participants with fewer retrospective than prospective scores (i.e., participants who had prospective measures of maltreatment types that they didn’t retrospectively self-report) were termed the ‘*omitted reports*’ group.

### 2.6. Qualitative analysis

We analysed the interviewer impression notes using the Framework Approach (Ritchie et al., 2003), comparing notes between groups to identify possible explanations for disagreement between prospective and retrospective measures of maltreatment. The Framework Approach was chosen for its systematic and flexible application of the principles of thematic analysis. The approach consists of six stages:

1. Data familiarisation: reading through the notes and writing down key ideas and themes emerging from the data.
2. Constructing a thematic framework: a structured coding scheme was established based on the themes and subthemes emerging from stage 1 and the study aim of exploring possible reasons for discrepancies between prospective and retrospective measures of maltreatment. The framework was refined through a collaborative review process with a second researcher and applied to a subset of the notes alongside the second researcher to test its applicability, compare interpretations, increase sensitivity, and ultimately move closer to the data.
3. Indexing: the thematic framework was systematically applied to the entire dataset using NVivo, assigning codes to data segments across all participants.
4. Charting: the indexed data were rearranged into thematic matrices in Excel, with themes and subthemes represented as columns and individual participants as rows. This enabled data associated with each participant to be easily navigated and analysed across different themes.
5. Mapping and interpretation: this was done by going backwards and forwards across the thematic matrices and back through the transcripts to develop the initial themes into higher level categories, and begin interpreting and making sense of the data to construct the final thematic framework. Themes were also refined through discussion with the second researcher.

## 3. Results

We analysed notes from interviews with 66 participants (hereafter referred to as the ‘study group’): 35 participants with greater retrospective than prospective maltreatment scores (the ‘new reports’ group), and 31 with greater prospective than retrospective maltreatment scores (the ‘omitted reports’ group). Notes ranged in length from 250 to 6919 words. The study group showed comparable gender distribution to the overall *E*-Risk Study cohort (48 % male in the study group vs. 49 % male in the overall sample). However, low socioeconomic status (SES) was over-represented (65 % in the study group vs. 33 % in the overall sample). In the ‘new reports’ group, 34 % of the participants were male, compared to 64 % of the ‘omitted reports*’* group, but low SES was similarly represented in both groups (63 % in the ‘new reports’ group vs. 67 % in the ‘omitted reports’ group). Table 2 summarises the demographic characteristics of participants in the ‘new reports’ and ‘omitted reports’ group.

Three key categories of themes emerged related to maltreatment measurement discrepancies (Table 3). The first category pertained to challenges with prospective measures of maltreatment, highlighting reasons given by the ‘new reports’ group (i.e., those with absent prospective measures) for why maltreatment was not detected or adequately responded to during prospective assessments at previous study phases. The second category related to challenges associated with measuring maltreatment through retrospective self-reports in terms of barriers to openness and accuracy in maltreatment disclosure, comparing notes across the ‘new reports’ and ‘omitted reports’ groups. The third focussed on differences that emerged between the two groups in their appraisals and interpretations of violence or distressing childhood experiences that might have led to either new or omitted retrospective self-reports.

### 3.1. Challenges with prospective measures: barriers to identifying and responding to maltreatment

The notes from participants with new retrospective reports of maltreatment highlighted the challenges of obtaining reliable information from prospective measures of maltreatment, including maltreatment being kept secret, ignored, disbelieved, or not adequately responded to by children’s services.

#### 3.1.1. Maltreatment kept secret

Notes from 12 interviews in the group with new retrospective reports revealed how participants kept maltreatment secret during their childhood and hence it was not identified in prospective caregiver interviews and official records. For one participant, the interview at age 18 was the first time she had disclosed maltreatment because she had had no one to confide in or seek help from. Other participants had hidden the physical signs of abuse, for example covering bruises and avoiding changing their clothes in front of people at school. Some participants had kept maltreatment secret because their abusers had “*threatened*” (*n* = 5) or *“blackmailed*” (*n* = 2) them. Notes from one interview described how a participant’s abusive father had kept her and her twin away from other relatives to hide the abuse. The participant had been too scared to tell anyone because of the threats from her father. Participants also described how their parents deliberately hid signs of abuse during previous *E*-Risk assessments:

“[The participant] said her mum used to put on a big show when [E-Risk Study interviewers] would come to visit in the past, but she did not mind as it was one time when Mum would be affectionate, and there would always be a hot dinner after as Mum would cook something to make it look like a perfect family.”

#### 3.1.2. Maltreatment ignored

Notes from six interviews revealed how caregivers ignored maltreatment and did not intervene:

“[The participant] was in so much pain after the beating and was covered in cuts and bruises, when they returned to Singapore [he] told Mum but she did nothing and Dad said he was lying. [The participant] felt betrayed by Mum.”

Participants described how they felt betrayed when their caregivers did not protect them. Three participants understood this to be because their caregivers chose to protect their abusive partner over their child. For example, one participant described to interviewers how she “*hated*” her mum “*taking [her boyfriend’s] side*” rather than keeping her daughter safe.

#### 3.1.3. Maltreatment not believed

Seven participants in the ‘new reports’ group described to interviewers how, as a child, they had disclosed the maltreatment to an adult but had not been believed. One participant told the interviewer that at age five she was sexually abused but her mother did not believe her until she was 14. Another participant described how he had tried to tell his stepfather, grandmother, and teacher about the maltreatment but *“wasn’t believed that it was going on to the extent it was* – *boys were being soft apparently”*.

#### 3.1.4. Lack of appropriate intervention by children’s services

In two cases, participants with new retrospective reports described instances where maltreatment went undetected, or no intervention occurred. One participant described how she had new bruises at school every week. She knew her teachers were suspicious, but they took no action to investigate. In another interview, the interviewer wrote:

“It seemed like the family ‘slipped through the net’ as many services knew there were ongoing problems with Mum but nobody intervened. The night [the participant] left home, it was because Mum had tried to strangle her on the stairs when she was drunk”

### 3.2. Challenges with retrospective measures: barriers to openness and accuracy in maltreatment disclosure

The notes across both groups also highlighted challenges and limitations associated with retrospective self-report measures, including distress as a barrier to disclosure, the importance of establishing rapport in the interview context, memory problems, and interviewers perceiving exaggeration of the reported experiences.

#### 3.2.1. Distress as a barrier to disclosure

Interviewers described how asking participants about maltreatment caused them distress and discomfort. One participant in the ‘new reports’ group explained how she hated talking about the sexual abuse she experienced as a child *“as she gets anxious and starts stuttering”.* In notes from interviews with four participants with omitted reports, interviewers described how they thought participants were withholding information because it was too upsetting or uncomfortable to discuss:

“[He] was quite uncomfortable in the [Juvenile Violence Questionnaire] and [Childhood Trauma Questionnaire] though – went bright red and was gripping his neck awkwardly when I mentioned about traumatic sexual experiences. However, he was very open and honest with me in the rest of the interview so other than that omission I think the interview was valid.”

In notes from eight interviews with the ‘new reports’ group, interviewers described how participants became emotional when asked about maltreatment, but were determined to tell “*the full story*” and found the process cathartic. Interviewers recognised the need for patience and sensitivity in these situations, and encouraged participants to take breaks if they became distressed. In contrast, other participants (*n* = 7) within this group were described as emotionally disconnected or “*matter-of-fact*” (*n* = 4) when discussing their experiences:

“I found her emotional numbness, facially/vocally and in description, unusual during the JVQ [Juvenile Victimization Questionnaire]. It seemed at times like she was telling me a list of facts rather than recounting personal events, such was her lack of emotion”.

#### 3.2.2. Establishing rapport

For eight participants with omitted retrospective reports, interviewers noted difficulties in eliciting information and participants’ answers being notably limited due to a perceived lack of rapport.

“Very difficult to establish any kind of rapport…Said that he was talkative with friends etc., so maybe he just didn’t feel comfortable with me. He was very quiet throughout the interview, and didn’t try to make conversation at any point. Didn’t elaborate at all and had to probe quite a lot to get details”.

Interviewers attributed this to participants’ shyness, withdrawal, disengagement or boredom, or general discomfort with the interview questions and environment. In three interviews with male participants, female interviewers reflected on how their gender may have made participants feel uncomfortable.

In contrast, in the ‘new reports’ group, 21 participants were described as easy to connect with: interviewers used adjectives such as “*open*” (*n* = 20), “*friendly*” (*n* = 14), and “*chatty*” (*n* = 5), and noted participants’ enthusiasm and gratitude for the opportunity to discuss their experiences (*n* = 11).

#### 3.2.3. Memory problems

In notes from nine interviews in the ‘omitted reports’ group, mentions of poor memory appeared, which might explain some cases of absent maltreatment reports. Some descriptions of poor memory were general, for example one participant explained he experienced memory loss due to cannabis use, while in another example the interviewer described:

“The twin had very poor memory, she could not recall where her GP surgery was, addresses where people live who she visits regularly, times and dates”.

Interviewers describe how participants appeared to struggle to remember when asked to recount distressing experiences. For example, one participant described how he only remembered *“bits and pieces”* about living with his Mum who had schizophrenia and died of an overdose when he was 11. Another explained why he had difficulty remembering a traumatic incident from his childhood:

“When talking about this, [he] said he could not remember exactly when it happened as he rarely talks about it and has always attempted to ‘block’ it out as though it never happened because he finds it hard to deal with”.

#### 3.2.4. Perceived exaggeration

In notes from three interviews in the ‘new reports’ group, interviewers expressed concerns about the consistency of participants’ accounts and perceived their descriptions of maltreatment as “exaggerated”. Interviewers perceived how their own behaviour and reactions might have influenced participants’ answers:

“She kept looking at me as though she was trying to judge my reaction as she told me more and more sort of shocking details… To be honest I just was thinking that she was making it up as she went along, I think she thought I believed her though and would continue to try to come out with ever more shocking stories… The more sympathy I expressed towards her the worse the details of the story would become”.

### 3.3. Different appraisals of distressing experiences

Aside from the challenges with prospective and retrospective measures noted above, differences between the participants with new reports and omitted reports emerged in their appraisals and interpretations of distressing childhood experiences. This included appraisal of intention and responsibility, perceptions of what is considered ‘normal’ and acceptable, and reflections on the long-term impact of distressing experiences.

#### 3.3.1. Appraisal of intention and responsibility

Different interpretations of intention and responsibility concerning dynamics between participants and their caregivers appeared in the notes. In notes from 20 interviews from the ‘new reports’ group, interviewers described how participants felt the maltreatment reflected a broader lack of love or care from their caregivers:

“[She] ran away from home after Mum hit her over the head with the stick of the hoover once. She was gone from home for about six months, but Mum only bothered to find out where she was after about five months, they had no contact up to then. She said it was a sign of how Mum feels about her”.

In contrast, in four interviews in the ‘omitted reports’ group, participants described violent relationships with their parents but did not report these as maltreatment. Instead, participants emphasised reciprocity, shared responsibility, or dismissed the severity of the incidents.

“She used to have ‘scraps’ with dad where they fought each other. She was never badly hurt but she was injured from the fights. He would hit her and vice versa.”

Another participant attributed the “*physical fights*” with her mother to the fact they had the “*same temperament*”.

#### 3.3.2. Appraisal of what is ‘normal’ and acceptable

Notes from 17 interviews with participants in the group with new retrospective reports revealed that participants came to recognise the maltreatment they were experiencing through comparison with the experiences of their siblings or friends.

“[The participant] does not feel Mum has parented him properly, does not treat the twins like he sees other people’s parents treating them… Does not feel his Mum and Stepdad, and Dad, are normal. ‘Not like other people’s parents’”.

Five participants described how they had gradually realised the maltreatment by challenging and reappraising their previous interpretations. In one interview with a participant whose mother regularly beat him, the interviewer noted:

“He thought [it] was just normal and it’s what mums did until a friend witnessed the abuse one day and told [him] that it wasn’t right, and his Mum shouldn’t be beating him”.

In contrast, in the ‘omitted reports’ group interviewers described how nine participants believed violent behaviour was normal or acceptable. One interviewer described the “very *blas*´*e* manner” with which a participant described her family’s violent behaviour. Participants also interpreted violence as “*justified*” or “*deserved*”, for example as retaliation to provocation or in the context of a fight. One participant whose uncle hit him justified the behaviour on the grounds that it was acceptable in their culture.

#### 3.3.3. Appraisal of the impact of distressing experiences

In 20 interviews with participants from the ‘new reports’ group, interviewers described how participants attributed problems in their current relationships and persistent behavioural or emotional issues to their history of maltreatment, and expressed a sense of being unable to move on. Appraisals of the continued impact of such experiences may increase the likelihood of participants choosing to report them. For some participants the consequences of maltreatment were evident only when participants recognised behaviour as abusive. For example, one participant explained his suicide attempt at age 12 was triggered when he realised he was being abused by his mother. For another participant, the impact of the abuse only fully manifested when it was acknowledged by her mother:

“[The participant] said she hated her mum for not believing her at age five and for ‘taking his side’ and said she almost tried to ignore it happened because of mum’s disbelief and not till age 14 when it all came out and mum finally believed did she let it affect her and accept that this really happened”.

Such descriptions and perceptions of the negative impact of distressing experiences were uncommon in those with omitted retrospective reports. Four participants explained to the interviewers how coping with stressful or difficult past experiences involved “*moving on*” and not being “*held back*” by negative memories. For three of these participants, the notes highlighted how they had positively reinterpreted difficult experiences from their past, for example:

“[The participant] said that his brothers were not in gangs but they hung around the wrong people and their friends were in gangs. He said that these events made him reflect, he has a strong belief that life is too short and can be taken away at any moment and therefore he is very strongly family orientated. [He] also felt that his family were lucky as both of his brothers survived being shot. [He] also decided that he didn’t want to go down the wrong path as he observed what the consequences of that can be”.

## 4. Discussion

An analysis of interviewer notes from E-Risk participants with discordant prospective and retrospective measures of maltreatment deepens our understanding of explanations for measurement disagreement. Our findings highlight difficulties associated with obtaining accurate and reliable information from caregivers as part of prospective measures, as well as challenges with asking individuals about their own maltreatment experiences retrospectively. The analysis also sheds light on the different ways in which the appraisal of maltreatment experiences can be influenced by individual, interpersonal, and wider cultural factors.

### 4.1. Comparison with existing research

Our finding that retrospective assessments capture first-hand accounts influenced by factors such as an individual’s recollection, reflection and processing of the abuse aligns with existing critiques of this measurement approach in the maltreatment literature (Hardt and Rutter, 2004; Widom et al., 2004). However, while prospective measures collected at the time are generally regarded as more valid than retrospective measures for gathering information on maltreatment (Li et al., 2016), our study highlights how they are limited by factors such as informant’s knowledge about and motivation to disclose maltreatment.

Our findings fit broadly within an existing framework that categorizes factors influencing maltreatment assessment into three broad domains: motivation, memory, and measurement (Baldwin et al., 2019; Danese, 2020). The findings support this framework by demonstrating how these factors interact to contribute to discrepancies in measures of maltreatment. They also caution against attributing maltreatment disagreement exclusively to a single domain, such as memory, instead underscoring the importance of considering multiple explanations existing simultaneously. The findings will be discussed in relation to existing research on motivation and memory, before exploring implications for the improvement of measurement.

With regard to motivation, contextual details in the notes reveal the complexities involved in the decision to disclose maltreatment, whether through prospective or retrospective assessments. Participants in the group with new retrospective reports described how the anticipated consequences of reporting maltreatment during childhood played a role in their decision to conceal maltreatment at the time, resulting in cases where trusted adults remained unaware of their experiences. They described the process of keeping maltreatment hidden as children, motivated by threats, blackmail or fear cultivated by the perpetrator, or feelings of shame or embarrassment in discussing abuse. Such motivations for concealing abuse are well documented in the clinical literature (Berliner and Conte, 1990; Paine and Hansen, 2002; Schaeffer, 1999) and research shows that victims of maltreatment commonly delay admitting maltreatment until adulthood (Bottoms et al., 2016; Goodman-Brown et al., 2003; Schönbucher et al., 2012). Prospective assessments involving caregiver interviews and official reports may not capture such concealed cases where children deliberately withheld their experience.

Our findings highlight how caregivers’ motivation to disclose abuse (or not) influenced prospective assessments, in cases where caregivers were aware of maltreatment but deliberately gave inaccurate or misleading information. The threat of mandatory reporting to child protection services if maltreatment is reported during childhood can lead to caregivers denying abuse due to concerns about the child being taken away, fear of family disintegration, or potential imprisonment of the perpetrator (Bottoms et al., 2016; Tajima et al., 2004). Social desirability bias may also influence caregivers’ decisions to report, leading them to downplay or deny maltreatment (Fisher et al., 2011). The *E*-Risk interviewer notes highlight situations where caregivers ignored signs of abuse or disbelieved their child, which further decreases the likelihood of the caregiver reporting abuse prospectively. Disbelief by mothers is associated with a closer relationship between mother and perpetrator at the time of disclosure (Everson et al., 1989). This is reflected in the E-Risk Study notes, where certain participants described feeling betrayed when their mothers defended the abusive partner.

The decision to report or withhold information about maltreatment when asked retrospectively is also shaped by individuals’ motivations at the time of the interview. Participants in both groups displayed signs of distress or discomfort when asked about maltreatment. In the group with omitted retrospective reports, participants’ unease during questioning led interviewers to suspect that they might be withholding information. The challenges of speaking out about experiences of maltreatment in childhood have been highlighted in previous qualitative work (Halvorsen et al., 2020), and feelings of shame, guilt and discomfort are common after maltreatment and well-documented barriers to disclosure (Lemaigre et al., 2017; McElvaney et al., 2022; Morrison et al., 2018).

The distress experienced by participants when discussing maltreatment underscores the importance of an interview environment where participants feel safe to discuss potentially distressing experiences (Femina et al., 1990). In the group with new retrospective reports, rapport was often easily established, with participants demonstrating a willingness to provide comprehensive accounts of their experiences. Conversely, in some cases in the group with omitted reports, interviewers encountered challenges in communication, resulting in limited responses and the impression of withheld information. These findings align with literature on forensic interview techniques highlighting the association between rapport and disclosure of abuse in both child and adult victims (Chenier et al., 2022; Femina et al., 1990; Hershkowitz et al., 2006, 2015). The findings also underscore the importance of interviewers being aware of their own positionality and power during interviews (e.g., potential gender, socioeconomic, age, and cultural hierarchies), including their perspectives, beliefs, or potential biases that might influence the research process and prevent certain participants from feeling comfortable discussing their experiences (Manohar et al., 2017).

With regard to memory process, their impact on retrospective self-reports of maltreatment has been discussed extensively (Danese, 2020; Hardt and Rutter, 2004; Maughan and Rutter, 1997; Susser and Widom, 2012). In the E-Risk notes, interviewers frequently noted participants’ difficulties in recalling childhood experiences, which is not uncommon in retrospective studies due to the extended time lapse between the events and reporting (Goodman et al., 2003; Greenhoot et al., 2005; Henry et al., 1994; Read and Widom, 1997). In the group with omitted reports, some participants spoke of having a generally poor memory, while others specifically struggled when trying to recall upsetting childhood experiences. While certain individuals may have better recall of negative memories compared to neutral events (Kensinger and Schacter, 2008), for others, negative memories can trigger avoidance strategies such as dissociation (Ehlers and Clark, 2000), cognitive avoidance (e.g., thought suppression) (Levy and Anderson, 2008), and over-general memory (Dalgleish et al., 2008). These avoidant strategies may lead some individuals to deliberately or non-deliberately avoid retrieving memories of maltreatment during interviews, leading to absent retrospective reports.

Our findings highlight how memories of maltreatment can be variously interpreted, labelled, and shaped by personal factors and broader cultural and social contexts, with consequences for self-report measures (Berger et al., 1988). Previous qualitative research indicates that factors such as perception of intention and sociocultural framing of parental behaviour as ‘normal’ or deviant, can influence individuals’ labelling of experiences as abuse (Aadnanes and Gulbrandsen, 2018). Self-blame has also been associated with reduced likelihood of disclosing an experience as abuse (Lemaigre et al., 2017).

Our findings also demonstrate shifting retrospective perspectives on maltreatment. Some participants in the group with new reports had realised that their experiences were not normal or acceptable as they grew older, while others assessed how these experiences had impacted their lives. In the group with new reports, many participants described how maltreatment had damaged their lives, whereas such descriptions were less common in the group with omitted reports. These variations in the appraisal of impact may contribute to differences in how participants conceptualized and remembered their experiences, reflecting the process of memory reappraisal (Alberini and LeDoux, 2013; Bartlett, 1932; Stige et al., 2020).

### 4.2. Implications for improving measurement of childhood maltreatment

Regardless of whether the measure of maltreatment is prospective or retrospective, it is imperative that studies incorporate comprehensive interviewer training addressing power, positionality, and bias. Furthermore, to aid in interpretating and contextualising the research findings, documentation of interviewer demographics, in conjunction with participant demographics, should be encouraged. For prospective measures, using multiple different informants and triangulating caregiver reports with other sources can help validate maltreatment reports and identify cases where caregivers are withholding information (e.g., Reuben et al., 2016). As illustrated by the *E*-Risk study, collaboration with GPs and social services to validate child protection concerns is one such example of this approach. The findings also highlight the critical role of responsive and proactive safeguarding services, and the need for children to be empowered and feel safe to disclose maltreatment, and to have a trustworthy and supportive adult to confide in. By focusing on these broad improvements in child welfare, the accuracy of prospective measures could also be increased.

Improving the measurement of retrospective self-reports of maltreatment requires addressing the challenges associated with discussing deeply personal and distressing experiences. Using a trauma-informed approach to qualitative research necessitates fostering a safe and confidential interview environment, establishing trust with the participant through active listening, empathy, and cultural sensitivity, and being aware of the risks of re-traumatization (Alessi and Kahn, 2023). Interviewers should receive comprehensive training to navigate any emotional distress caused by conversations about maltreatment. Understanding and sensitively addressing emotions such as shame, guilt, and distress can also foster an atmosphere of trust, enabling individuals to share experiences more openly and honestly (Deblinger and Runyon, 2005). Offering study participants the option to use written questionnaires rather than interviews, a technique shown to encourage greater openness in certain studies (Kim et al., 2008), could alleviate the difficulties or distress associated with verbalising certain experiences.

Finally, considering the nuanced meanings and interpretations individuals give to their experiences may offer an important avenue for improving self-report measurements of maltreatment. Furthermore, exploring the nature and organization of maltreatment memories may provide deeper insights into differences in the way individuals subjectively experience and remember maltreatment (Bifulco and Schimmenti, 2019). This, in turn, could improve our understanding of why subjective self-reports of maltreatment are more strongly associated with psychopathology than objective measures (Baldwin et al., 2024; Danese and Widom, 2020)

### 4.3. Strengths and limitations

A key strength of this study is its use of a longitudinal cohort with repeated measures of maltreatment collected over childhood and adolescent years. By conducting interviews and collecting data over a prolonged period, E-Risk Study interviewers had the opportunity to establish trust and familiarity with participants and their caregivers throughout various phases of the study. The use of repeated measures collected over time also increases the sensitivity of the prospective measures. In addition, the large overall sample size of this cohort facilitated a diverse sampling strategy, enabling the selection of a subset of participants with the most discrepant reports. The involvement of a large team of interviewers (*N* = 14) also mitigates the impact of individual biases that would have been more pronounced with a smaller group of interviewers. However, evaluation by multiple different interviewers may also add measurement error due to variations in techniques and interpretations.

In addition, as the notes were not initially intended to be used for qualitative interpretation, they are more naturalistic and less susceptible to the potential biases that might arise when interviewers are aware that their impressions and notes will undergo analysis. However, using data that was not originally collected for the purpose of qualitative analysis also has limitations, as interviewers did not follow a specific protocol for note-taking after the interviews. While all interviewers received the same training, this lack of standardized procedure inevitably results in variations in the focus, volume, and relevance of information captured in the notes. Future research could address this using more focused data collection methods.

The perspective provided by the interviewers’ notes also presents both strengths and limitations. On the one hand, it provides valuable insights into the processes and challenges of retrospective maltreatment assessment from the assessor’s perspective, therefore pointing to areas for potential improvement in measurement methods. On the other hand, it does not capture first-hand perspectives of individuals reporting maltreatment; instead, their accounts are conveyed through the intermediary lens of the interviewer. Further research that incorporates first-person perspectives from individuals detailing their experiences of the interview process and childhood narratives should be prioritised.

Finally, because of the limited number of participants with prospective or retrospective measures of maltreatment and our a-priori decision to focus on those with the most discordant scores across the sample, excluding one twin from each pair would have been an overly restrictive sampling strategy to identify the participants for qualitative analyses. However, the twin design and family relatedness of some participants might have biased the themes identified. With regard to generalisability, we observe that the prevalence of maltreatment observed in this cohort closely mirrors contemporary rates within the broader population of children (Radford et al., 2013).

### 4.4. Future directions

As highlighted in the limitations, our study used secondary data from a cohort study not originally designed for qualitative analysis. Therefore, to gain deeper insights into the complex dynamics of underlying disagreement between maltreatment measures, future research should utilize more targeted study designs.

Firstly, our cumulative approach, based on meta-analytic findings that agreement between maltreatment measures is broadly invariant across different maltreatment subtypes (Baldwin et al., 2019), did not allow for the comparison of specific maltreatment subtypes. Future studies could adopt targeted recruitment strategies to more clearly map themes related to disagreement between maltreatment subtypes.

Secondly, targeted recruitment of male and female samples or a gender-balanced sample would facilitate a more detailed description of themes that differentially relate to either gender.

Thirdly, while our analysis identified a set of themes, it is unlikely that these themes operate in isolation. Future research should employ designs that explore the intersections of these themes, such as focused interview questions that investigate how participants’ explanations overlap. Exploring how different themes interact could offer a more comprehensive understanding of the dynamics influencing discrepancies in maltreatment measures.

## 5. Conclusions

This qualitative study points to the limitations of both prospective and retrospective measures of childhood maltreatment, and the different underlying constructs they capture. Prospective measures provide a third-person perspective through informants, influenced by factors such as their knowledge of events, definitions of maltreatment, and motivations to disclose. On the other hand, retrospective measures offer a first-person, subjective view from respondents, shaped by their motivations, appraisals, and memory processes. Recognising these distinct constructs and their current limitations is crucial for enhancing measurement strategies and better identifying and helping individuals who have experienced maltreatment.

## Supplementary Material

supplemental document

Supplementary data to this article can be found online at https://doi.org/10.1016/j.chiabu.2024.107070.

## Figures and Tables

**Table 1:** E-Risk participants categorized by maltreatment discrepancy score.

Discrepancy score	*n* (%)
−4	0 (0)
−3	6 (0.3)
−2	25 (1.2)
−1	90 (4.4)
0	1786 (86.9)
1	113 (5.5)
2	26 (1.3)
3	7 (0.3)
4	2 (0.1)
Total	2055

*Note.* A score of “0” indicates that participants had the same number of maltreatment types measured prospectively and retrospectively. Scores below “0” indicate that participants had a greater number of prospectively identified maltreatment types than they retrospectively reported, with lower scores indicating greater discrepancy between measures. Scores above “0” indicate that participants retrospectively reported a greater number of maltreatment types than were prospectively identified, with higher scores indicating greater discrepancy between measures.

**Table 2:** Sociodemographic characteristics of discrepant maltreatment reporters (*N* = 66).

		‘*New reports’* group n (%)	‘*Omitted reports’* group n (%)
Participants		35	31
Twin pairs included		5	10
Sex	Male	12 (18.2)	20 (30.3)
	Female	23 (34.8)	11 (16.7)
SES	Low	22 (33.3)	21 (31.8)
	Medium	11 (16.7)	6 (9.1)
	High	2 (3)	4 (0.1)

*Note.* SES, family socioeconomic status at age 5. The ‘*new reports’* group consisted of participants who self-reported types of maltreatment that were not prospectively detected. The ‘*omitted reports’* group consisted of participants who had prospectively measured maltreatment types that they didn’t retrospectively self-report.

**Table 3:** Thematic framework exploring potential sources of disagreement between prospective and retrospective measures of maltreatment.

Theme category	Theme	Definition
Challenges with prospective measures	Maltreatment kept secret	Instances where participants described how they or their parents had intentionally concealed experiences of maltreatment during childhood
	Maltreatment ignored	Cases where participants described that their caregivers were aware of maltreatment but choose to overlook or dismiss it
	Maltreatment not believed	Situations in which participants described facing scepticism or disbelief regarding maltreatment they had disclosed to caregivers or other adults
	Lack of appropriate intervention by children’s services	Cases where maltreatment went undetected or was not adequately responded to by child protective services, as described by participants
Challenges with retrospective measures	Distress as a barrier to disclosure	Emotional discomfort or distress experienced by participants when asked about maltreatment during interviews
	Establishing a rapport in the interview context	Descriptions of the level of rapport built between the interviewer and participant during the interview
	Memory problems	Descriptions of participants having difficulty recalling specific details or experiences due to memory constraints
	Perceived exaggeration	Instances where interviewers expressed concern about participants possibly inflating or creating false accounts of maltreatment
Different appraisals of distressing experiences	Appraisal of intention and responsibility	Participants’ assessments of the motives and accountability behind certain behaviours that might influence their judgement on what constitutes maltreatment
	Appraisal of what is ‘normal’ and acceptable	Participants’ interpretations of personal or cultural norms that might influence their judgement of whether experiences constitute maltreatment
	Appraisal of the impact of distressing experiences	Participants’ evaluations of how certain distressing experiences have affected their lives

## Data Availability

The data that has been used is confidential.
