# Supplementary material for: Why do prospective and retrospective measures of childhood maltreatment differ? Qualitative analyses in a cohort study
Source: Child Abuse Negl. Author manuscript; Available in PMC 2025 Jun 5. (PMC12139143; doi:10.1016/j.chiabu.2024.107070)
Supplement: supplemental document [file NIHMS2077462-supplement-supplemental_document.docx]

**Supplementary Materials**

**Prospective measures of childhood maltreatment**

Exposure to several types of maltreatment was assessed repeatedly when the children were 5, 7, 10, and 12 years of age and dossiers have been compiled for each child with cumulative information about exposure to physical abuse by an adult; sexual abuse; physical neglect; and emotional abuse/neglect. The E-Risk team has previously reported evidence on the reliability and validity of the measures of physical maltreatment and sexual abuse ([Jaffee, Caspi, Moffitt, & Taylor, 2004](#_ENREF_25)), emotional abuse/neglect (Danese et al., 2017), and physical neglect (Fisher et *al*., 2015). All the component measures are outlined briefly below.

*Physical and sexual harm by an adult.* We assessed childhood physical and sexual harm in the E-Risk Study using an approach that resembles the process undertaken by child protection agencies. Essentially this is a two-stage process. In child protection, professionals such as teachers working with children typically raise concerns if they observe signs or symptoms or if they become aware of risk that children are victims of violence. When concerns are raised, child protection officers then review the concerns and evaluated them in the context of information previously gathered on that child or family in order to determine the likelihood that abuse has taken place. In the E-Risk Study, research workers visited the home in pairs, and were extensively trained to detect signs of abuse or neglect. Each time the two research workers visited a home, they interviewed the mother using a structured interview about child harm, tested the children, and observed the family environment using the Home Observation for Measurement of the Environment (HOME) (Bradley and Caldwell, 1977). If either research worker had any concerns, they flagged up the case for review. Immediately after each home visit, a review was performed if a family was flagged. In addition, at each wave, any family who had been flagged on a prior wave of the study was automatically reviewed again. The reviews were performed independently by at least 2 clinical psychologists or psychiatrists, and were based on comprehensive dossiers compiled across multiple home visits for each study member during the course of the ongoing longitudinal study.

An unusual feature of the E-Risk study’s assessment is that we repeatedly interviewed mothers on four occasions over the years, which allowed them to build confidence in the research team. Also, we were able to reassure mothers that if harm to the child was ongoing and had to be reported by us, reporting would be managed through a trusted familiar professional, namely the family’s registered GP. As the children grew older, some mothers who were initially reluctant to reveal abuse to us, divulged details of severe abuse at a later interview.

At age 5, assessments were based on the standardised clinical protocol from the MultiSite Child Development Project (Dodge, Bates, & Pettitt, 1990; Lansford *et al*., 2002). At ages 7, 10, and 12 this interview was modified to expand its coverage of contexts for child harm. Interviews were designed to enhance mothers’ comfort with reporting valid child maltreatment information, while also meeting researchers’ responsibilities for referral under the UK Children Act. Specifically, mothers were asked whether either of their twins had been intentionally harmed (physically or sexually) by an adult or had contact with welfare agencies. If caregivers endorsed a question, research workers made extensive notes on what had happened, and indicated whether physical and/or psychological harm had occurred. Under the UK Children Act, our responsibility was to secure intervention if maltreatment was current and ongoing. Such intervention on behalf of E-Risk families was carried out with parental cooperation in all but one case. No families left the study following intervention.

Over the years of data collection, the study developed a cumulative profile for each child, comprising the caregiver reports, recorded debriefings with research workers who had coded any indication of maltreatment at any of the successive home visits, recorded narratives of the successive caregiver interviews, and information from clinicians whenever the Study team made a child-protection referral. Each time we visited a home, the research workers flagged concerns, and if there was sufficient evidence to code definite harm then we did so. If evidence only met the level of probable harm, we kept an “ongoing concern list” and if, at a later wave, there was continued evidence of probable harm, or new evidence, the code was upgraded to definite harm. The profiles were reviewed at the end of the age-12 phase by at least two clinical psychologists or psychiatrists. Initial inter-rater agreement between the coders was 90% in cases for whom maltreatment was identified (100% for cases of sexual abuse), and discrepantly coded cases were resolved by consensus review. These were coded as: 0 = no physical harm at any age; 1 = probable physical harm at any age; and 2 = definite physical harm at any age. When limited to the 2055 participants with CTQ data, there were 12.5% of children coded as probably being exposed to physical harm and 7.4% as definitely physically harmed by 12 years of age. There were 1.6% of the children coded as probably or definitely being exposed to sexual abuse.

*Physical neglect.* The cumulative observations of the physical state of the home environment documented by the research workers during home visits to the twins at ages 5, 7, 10 and 12 were reviewed by two raters for evidence of physical neglect. This was defined as any sign that the caretaker was not providing a safe, sanitary, or healthy environment for the child. This included the child not having proper clothing or food, as well as grossly unsanitary home environments (However, this did not include a family living in a crime-ridden neighborhood for economic reasons). Inter-rater agreement between the coders exceeded 85%, and discrepantly coded cases were resolved by consensus review. When limited to the 2055 participants with CTQ data, there 7.3% children with indication of minor physical neglect (coded 1), and where there were 1.7% children with indication of more severe physical neglect (coded 2).

*Emotional abuse and neglect.* These forms of maltreatment were coded from research workers’ narratives of the home visits at ages 5, 7, 10, and 12. We coded quite severe examples of parental behavior observed. For example, a mother who had schizophrenia screamed and swore at the children throughout the home visit. As another example, a father who was drunk during the home visit repeatedly spoke abusively to the children in front of the research workers. We found that coders could not empirically separate emotional abuse and emotional neglect in a reliable way and thus such experiences were coded together as emotional abuse/neglect. Inter-rater agreement between the coders exceeded 85% for cases with emotional abuse and neglect, and discrepant cases were resolved by consensus review. Children with no evidence of emotional abuse/neglect were coded as 0 (88.5%), those where there was some indication of emotionally inappropriate/potentially abusive or neglectful behavior were coded as 1 (8.5%), and where there was evidence of severe emotional abuse/neglect the children were coded as 2 (3.0%).

Supplementary Figure 1. The E-Risk Study families’ addresses are a near-perfect match to the deciles of the UK government’s Index of Multiple Deprivation.

*Note.* This histogram shows E-Risk families’ addresses are a near-perfect match to the deciles of the UK’s 2015 Lower-layer Super Output Area (LSOA) Index of Multiple Deprivation (IMD) which averages 1,500 residents (or 650 households each); approximately 10% (dotted red line) of the E-Risk cohort fills each of the IMD’s 10% bands, indicating that the E-Risk cohort accurately represents the distribution of deprivation in the UK.

**References**

Bradley, R., & Caldwell, B., 1977. Home observation for measurement of the environment: a validation study of screening efficiency. **Am. J. Ment. Defic**. 81 (5), 417-420.

Danese, A., Moffitt, T.E., Arseneault, L., Bleiberg, B.A., Dinardo, P.B., Gandelman, S.B., Houts, R., Ambler, A., Fisher, H.L., & Poulton, R., 2017. The origins of cognitive deficits in victimized children: implications for neuroscientists and clinicians. Am. J. Psychiatry 174 (4), 349-361.

Dodge, K.A., Bates, J.E., & Pettit, G. S., 1990. Mechanisms in the cycle of violence. Science 250, 1678-1683.

Fisher, H.L., Caspi, A., Moffitt, T.E., Wertz, J., Gray, R., Newbury, J., Ambler, A., Zavos, H., Danese, A., Mill, J., Odgers, C.L., Pariante, C., Wong, C., C, Y., & Arseneault, L., 2015. Measuring adolescents' exposure to victimization: The Environmental Risk (E-Risk) Longitudinal Twin Study. Dev. Psychopathol. 27 (4pt2), 1399-1416.

Jaffee, S.R., Caspi, A., Moffitt, T.E., & Taylor, A., 2004. Physical maltreatment victim to antisocial child: evidence of an environmentally mediated process. J. Abnorm. Psychol. 113 (1), 44-55.

Lansford, J.E., Dodge, K.A., Pettit, G.S., Bates, J.E., Crozier, J., & Kaplow, J., 2002. Long-term effects of early child physical maltreatment on psychological, behavioral, and academic problems in adolescence: a 12-year prospective study. Arch. Pediatr. Adolesc. Med. 156 (8), 824-830.
